# Price regulation, new entry, and information shock on pharmaceutical market in Taiwan: a nationwide data-based study from 2001 to 2004

**DOI:** 10.1186/1472-6963-10-218

**Published:** 2010-07-25

**Authors:** Fei-Yuan Hsiao, Yi-Wen Tsai, Weng-Foung Huang

**Affiliations:** 1 Pharmaceutical Health Services Research Department, University of Maryland School of Pharmacy. Baltimore, MD21201, USA; 2 Institute of Health and Welfare Policy, National Yang-Ming University, Taipei, Taiwan; 3 Institute of Population Health Sciences, National Health Research Institutes, Miaoli, Taiwan

## Abstract

**Background:**

Using non-steroidal anti-inflammatory drugs (NSAIDs) as a case, we used Taiwan's National Health Insurance (NHI) database, to empirically explore the association between policy interventions (price regulation, new drug entry, and an information shock) and drug expenditures, utilization, and market structure between 2001 and 2004.

**Methods:**

All NSAIDs prescribed in ambulatory visits in the NHI system during our study period were included and aggregated quarterly. Segmented regression analysis for interrupted time series was used to examine the associations between two price regulations, two new drug entries (cyclooxygennase-2 inhibitors) and the rofecoxib safety signal and expenditures and utilization of all NSAIDs. Herfindahl index (HHI) was applied to further examine the association between these interventions and market structure of NSAIDs.

**Results:**

New entry was the only variable that was significantly correlated with changes of expenditures (positive change, p = 0.02) and market structure of the NSAIDs market in the NHI system. The correlation between price regulation (first price regulation, p = 0.62; second price regulation, p = 0.26) and information shock (p = 0.31) and drug expenditure were not statistically significant. There was no significant change in the prescribing volume of NSAIDs per rheumatoid arthritis (RA) or osteoarthritis (OA) ambulatory visit during the observational period. The market share of NSAIDs had also been largely substituted by these new drugs up to 50%, in a three-year period and resulted in a more concentrated market structure (HHI 0.17).

**Conclusions:**

Our empirical study found that new drug entry was the main driving force behind escalating drug spending, especially by altering the market share.

## Background

Over the past decades, the worldwide pharmaceutical market has become characterized by persistent increase in expenditures [1]. This has attracted the attention of policymakers and provoked questions about trends and factors in the unending escalation of pharmaceutical spending [2,3]. The three main components typically identified as affecting pharmaceutical spending are the effects of price, volume and therapeutic choice [2,4,5]. Health policymakers believe that these components are, in turn, primarily affected by policy interventions such as drug price regulation or reimbursement of new technology [3]. Despite the large assumed importance of these policy interventions, very little objective data is actually available about the extent to which these interventions influence the drug market.

Drug price regulation policies have been investigated in previous studies, although the association between price regulation and drug spending is questioned [6,7]. Although theory has suggested that drug market shift due to price regulation could be a significant confounder in assessing the controversial effects of price regulation on cost containment [8], no product-level data has ever been collected to examine market redistribution from product substitution post-price regulation.

Similarly, existing evidence regarding new technologies usually focuses on their potential economic burden but ignores their market impact. New technology entries are never a single market event. Instead, new technologies diffuse into the market. As a result, it is important to establish a longitudinal evaluation of the diffusion of new drugs into a medical care system. This allows policy makers to monitor patients' access to new drugs and contain unnecessary expenditures. However, unlike innovation within other markets [9-12], studies on the diffusion patterns of new drug are relatively limited. In addition, post-marketing information of new drugs [13] may have influence on drug market but have limited empirical data as well. Since the specific policies that would be implemented to curb rising costs would differ based on the source of expenditure increase, it is important to examine whether expenditure changes are attributable to price regulation, new entry, or post-marketing information.

The purpose of this study, therefore, was to use Taiwan's National Health Insurance (NHI) database, to empirically explore the association between policy interventions (price regulation, new entry, and information shock) and drug expenditure, utilization, and market structure across time frame. For the purpose of this study, the particular pharmaceutical market we chose was that of the non-steroidal anti-inflammatory drugs (NSAIDs) and cyclooxygennse-2 (COX-2) inhibitors, and the time frame was a 4-year time period, 2001-2004.

## Methods

### Data source

Our data were drawn from the 2001-2004 NHI databases, a nationally, population-based claims database. There're several advantages of using Taiwan's NHI database [14] to quantify the changes of drug expenditure, utilization and market structure after policy interventions. First, this mandatory health insurance program, with approximately 23 millions insured, covers nearly over 99% population of Taiwan. The enrollees of this program are predominantly employer-based but also include disadvantaged individuals, such as people in the low-income or disability sectors. This database thus allows policy makers as well as researchers to trace the changes of national drug expenditure and market structure after implementing these interventions in a closed medical system.

Second, under the single-payer system of NHI, Taiwan has established a national formulary (positive list), which includes all drug products (~21,000 products) subject to reimbursement by NHI. This detailed list of drug formulary allows the researcher to provide information on prescriptions of each NSAIDs product dispensed to their beneficiaries and associated cost paid by the NHI at the level of product.

### Identifying policy interventions in this study

We provided quarterly data between January 2001 and December 2004 on all our analyses. In our analyses, we aimed to track two price regulations that could affect the use and costs of NSAIDs: the price regulation implemented in April 2001 (the second season (S2) of 2001; 2001S2) and March 2003 (2003S1). In, Taiwan, the NHI imposes direct price controls on drugs by fixing the reimbursement prices product by product. Every one or two year, the NHI implements the price regulation to re-set (usually decrease) the reimbursement price of each product. The association between NHI's reimbursement of two COX-2 inhibitors, the entries of these new NSAIDs, in April 2001 (2001S2) and July 2001 (2001S3) and changes of drug expenditure were also of concern. According to NHI's Principles on Drug Reimbursement Price Approval [15], a new drug is defined as a newly applied pharmaceutical product that owns a new chemical entity, new dosage form, new administrated route or new therapeutic effect compound to the listed items in the pharmaceutical benefit scheme. New drugs are further categorized as breakthrough, me-too, and line extension based on drug innovation. Different reimbursement price policies are applied in accordance with how drugs are categorized based upon these definitions. Both of the COX-2 inhibitors were defined as breakthrough new drugs and were reimbursed by the NHI at a relatively high price (celecoxib 100 mg, NT 16.7 and rofecoxib 25 mg, NT 33.4;, currency exchange rate is approximately NT33 to US1) as compared to non-selective NSIADs (mean price per tablet ~ NT 3.00) upon approval. Finally, the voluntary withdrawal of rofecoxib worldwide on September 30, 2004 (2004S3) due to an increased cardiovascular risk, two years after it had been introduced into the market, was considered as an information shock in our study.

### Analyses

All NSAIDs prescribed in ambulatory visits in the NHI system during our study period were included. In Taiwan, NSAIDs could be prescribed by physicians to patients who needed it (approved indications from common pain to arthritis) and covered by the NHI program, patients could then get NSAIDs without out-of-pocket payment. As a result, the NHI database could capture the use and cost of NSAIDs.

For each NSAIDs prescribed, the pharmacy record of NHI databases included drug codes (a 10-digit coding system that uniquely identifies product reimbursed by the NHI), dosages, quantities, starting date (date prescription was dispensed) and prescription duration to track all necessary information. For those who took any of these medicines, the NHI databases also provided information allowing one to track the indications of their treatment.

Firstly, we calculated the aggregated mean quarterly drug cost (reimbursement price multiply quantities filled) and volume (converted into defined daily dose (DDD)) according to the World Health Organization definitions [16]) of all NSAIDs products. Drug cost and volume of NSAIDs per ambulatory visit were then provided. We further specified drug cost and volume of NSAIDs per rheumatoid arthritis (RA) or osteoarthritis (OA) ambulatory visit since they are two main indications of NSAIDs.

Segmented regression analysis for interrupted time series was used to determine the significance of the differences in slopes over time due to four interventions: (1) price regulation I (April, 2001), (2) new entry (July, 2001), (3) price regulation II (March, 2003), and information shock (September, 2004). While the pre-intervention segment serves as the control for the post-intervention segment, segmented regression analysis for interrupted time series data provides a credible methodological approach to measure the effect attributable to a specific event in time, i.e. the implementation of an intervention [17]. Proper estimations of standard errors and significance were made through the detection of and correction for autocorrelation. The Durbin-Watson statistic was used to test for autocorrelation. Seasonal effect of our data was also tested with the Dickey-Fuller unit test. Significance was determined at the 0.05 level, and SAS 9.1 (SAS, Cary, NC) was used for the statistical analyses.

In order to describe market share of all NSAIDs products over time, we categorized these products based on their reimbursement price. For those prices in 75%, 25-75%, and 25% quartile of total products, we defined them as high-priced, medium-priced, and low-priced NSAIDs, respectively. The category was then used to describe the change of market share of NSAIDs during our study period. In addition, we separated two COX-2 inhibitors from high-priced NSAIDs to further clarify their net effect as a new technology on change of market share of NSAIDs. We then applied Herfindahl index of concentration (the sum of the squared market shares of all products) to further estimate the effect of policy interventions on market structure of NSAIDs over time. In general, Herfindahl indices between 0.10 and 0.18 define the market to be moderately concentrated and indices above 0.18 to be concentrated. As the market concentration increases, competition decrease and the chances of monopoly increase.

## Results

**Figure 1:**
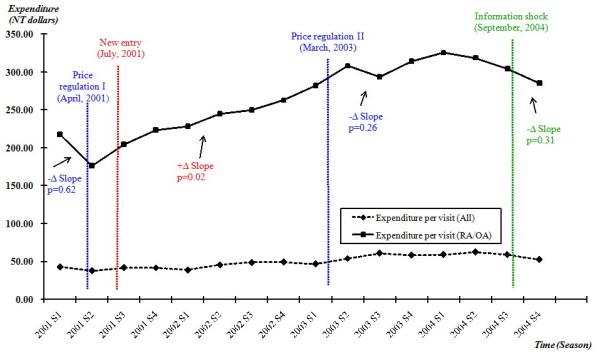
**Expenditure of NSAIDs per RA/OA ambulatory visit, quarterly, 2001-2004**. Quarterly expenditure of NSAIDs per RA or OA ambulatory visit was plotted with the timing of each policy intervention marked. Quarterly expenditure of NSAIDs per ambulatory visit (for any ambulatory visit with NSAID prescription) was also plotted separately as a reference group. The mean quarterly cost of NSAIDs per RA or OA ambulatory visit increased approximately 30% at the end of 2004 following the reimbursement of two new drugs (COX-2 inhibitors).

Cost of NSAIDs per RA or OA ambulatory visit varied over the period of study. The mean quarterly cost increased approximately 30% from NT 217.40 to NT 285.40 (currency exchange rate is approximately NT33 to US1) at the end of 2004. The cost dropped immediately by almost 20% after the price regulation was introduced in April 2001 (2001S2), although the negative change was not significant (p = 0.62). This decrease was short-lived, however, after the adoption of two COX-2 inhibitors. The cost has significantly increased 40% after adopting the COX-2 inhibitors (positive change, p = 0.02). The second price regulation did not stop the trend (negative change, p = 0.26). However, the expenditure declined when rofecoxib, one of the new entries, was withdrawn from the market (negative change, p = 0.31) (Figure 1).

**Figure 2:**
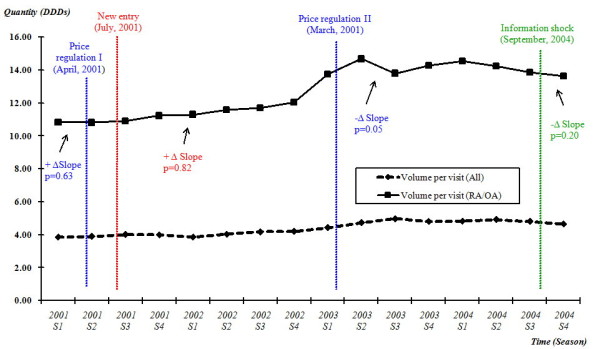
**Volume (DDDs) of NSAIDs per RA/OA ambulatory visit, quarterly, 2001-2004**. Quarterly prescribing volume of NSAIDs per RA or OA ambulatory visit was plotted with the timing of each policy intervention marked. Quarterly prescribing volume of NSAIDs per ambulatory visit (for any ambulatory visit with NSAID prescription) was also plotted separately as a reference group.

There was no significant change in the prescribing volume of NSAIDs per RA or OA ambulatory visit, even after the implementation of first price regulation (p = 0.63) and adoption of two COX-2 inhibitors (p = 0.82). A 7% increase of NSAIDs volume, from 13.74 DDDs (2003 S1) to 14.68 DDDs (2003 S2) was observed sooner after the introduction of the second price regulation in March 2003 (2003 S1) indicating a higher utilization in response to the lower price reimbursed. However, this change was short-lived because the long-term change after the implementation of second price regulation was not statistically significant (p = 0.05) (Figure 2).

**Figure 3:**
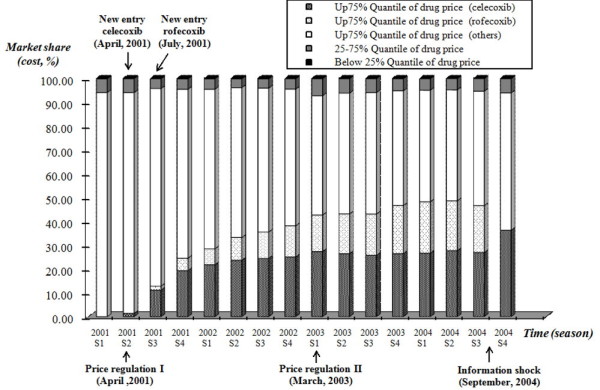
**Market share of NSAIDs products, quarterly, 2001-2004**. The market share (cost) of NASIDs products had been largely substituted by celecoxib and rofecoxib up to 26.92% and 19.68%, respectively, in a three-year period.

The change of the NSAIDs market structure for RA/OA treatment after new entries was of concern. Although NSAIDs is a crowded therapeutic class, choices of NSAIDs for RA/OA treatment had been mostly from high-priced products (about 95% of NSAIDs prescriptions consumed) across our observational period. This NSAIDs market, however, was changed after the introduction of two COX-2 inhibitors (celecoxib and rofecoxib). The market share of these products had been largely substituted by celecoxib and rofecoxib up to 26.92% and 19.68%, respectively, in a three-year period (from 2002 S2 to 2004 S3). Celecoxib, the pioneer COX-2 inhibitor, appeared to have the first-mover advantage in Taiwan's NHI system (Figure 3).

**Figure 4:**
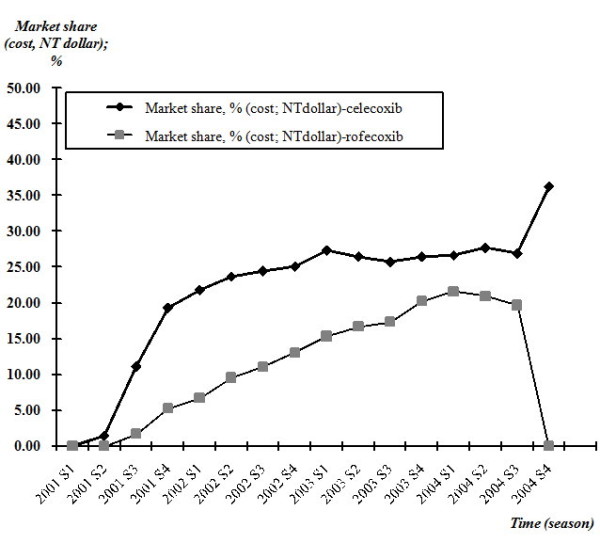
**Diffusion pattern of two COX-2 inhibitors (market share; cost (NT dollars)), quarterly, 2001-2004**. The market share (cost) of celecoxib in the NSAIDs market rapidly increased to 19.32% in only six months (2001 S4) after its listing into the NHI's benefit coverage and continued to increase thereafter. Its competitor, the follower COX-2 inhibitor (rofecoxib), however, took about two years to reach its market share (cost) to 20.22% (2003 S4) after its listing and sustained a 5% market share gap to celecoxib thereafter.

The market share of celecoxib rapidly increased to 19.32% in only six months (2001 S4) after its listing into the NHI's benefit coverage and continued to increase thereafter. Its competitor, the follower COX-2 inhibitor (rofecoxib), however, did not follow the same diffusion pattern as celecoxib. It took about two years to reach its market share to 20.22% (2003 S4) after its listing and sustained a 5% market share gap to celecoxib thereafter. Overall, the combined effect of new entries had taken about 50% (2004 S3) of the market originally taken by other high-priced NSAIDs. The information shock due to rofecoxib's withdrawal, yet, was a good opportunity to celecoxib (market share of celecoxib from 26.92% to 36.30%) and other high-priced NSAIDs (market share from 48.01% to 57.60%) because they immediately took the market originally taken by rofecoxib (Figure 4).

**Figure 5:**
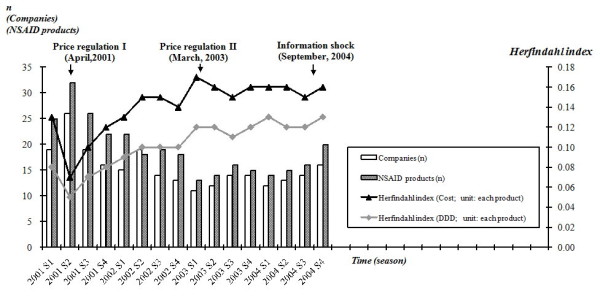
**Market structure of major market of NSAIDs for RA/OA treatment, quarterly, 2001-2004**. Number of products and drug companies that contributed to the top 80% NSAIDs market was plotted across time. Herfindahl index of concentration (HHI) was also provided to further estimate the effect of policy interventions on market structure of NSAIDs over time.

Figure 5 displays the major market of NSAIDs for RA/OA treatment (major market was defined as the market that comprised NSAIDs products which account for the top 80% of market share (cost)) and applies Herfindahl index of concentration (HHI) to further estimate the effect of interventions on market structure of NSAID over time. To our surprise, only 25 products in 19 pharmaceutical companies involved in the major market we're interested in (2001 S1). In other words, 80% of the cost of NSAIDs for RA/OA treatment was consumed by very limited NSAIDs products (25 in 660 (3.79%) products reimbursed by the NHI). Although the market structure has once become unconcentrated after the first price regulation it has became more and more concentrated since the utilization of NSAIDs had been significantly substituted by COX-2 inhibitors. At the end of 2002, less than two years after the adoption of COX-2 inhibitors, only 18 products in 13 pharmaceutical companies remained in the major market. In the short run, information due to rofecoxib's withdrawal did not change the major market of NSAIDs for RA/OA treatment.

## Discussion

Pharmaceutical health policies are designed to regulate cost, access and quality of patented drugs. Many of the assumptions upon which policies are designed, however, have not been empirically substantiated. This study was conducted to provide a national longitudinal trend of the NSAIDs pharmaceutical market in Taiwan during the 4-year period with events in three kinds of interventions (i.e., price regulation, new entries and information shock). In the entire 4-year period drug cost per RA/OA visit increased approximately 30% and we have found that new entry could be the main driving force that impacts the pharmaceutical market most in Taiwan even under policies of price regulation. The fact that market entry of COX-2 inhibitors was associated with nearly a one-third increase in the cost per visit raises concerns about the comparative value of new drugs, especially in therapeutic area where some might question whether the new drugs are truly distinguished from older therapies.

This study captured the market changes from two price regulations implemented by the Bureau of NHI. However, the association between these regulations and the market changes was very short-lived, especially under the driving force of entries of new COX-2 inhibitors. In Taiwan, price regulations were based on results of an annually survey of market price and volume. The "reference pricing" or "generic grouping" techniques were also used to reduce the price variation among products with similarity of active ingredients. Using 1996-2003 NHI's claim data, Lee et al [18] has reported that "reference pricing" or "generic grouping" used in the price regulation schemes on April 2001 and March 2003 were the most effective price control strategies for reducing total drug spending in Taiwan. However, in the study of Lee et al [18], the impact of price regulation on the total pharmaceutical spending appeared to be short-lived as observed in our study.

This study, using NSAIDs as a case, has tried to reveal a more detailed picture of changes in drug expenditure, utilization, and market structure after price regulations. Although the two price regulations used similar concepts and techniques, their impacts are different. The second price regulation implemented on March, 2003 (2003S1), however, did not significantly changes NSAIDs expenditure. Instead, it was associated with a substantial change of the NSAIDs volume prescribed for RA/OA treatment. Market redistribution may have resulted from providers' replacing products under price regulation with other more profitable products. Alternately, the resulting prescription volume increases may have canceled the changes due to price regulation in a short period.

Of great value, this study elucidates how new drugs diffuse into the medical care system, how they begin to substitute for existing products, and how they change the cost of treatment. It is evident from our empirical analysis that the first new entry, celecoxib, diffused rapidly and took less than one year to reach to its plateau of market share (23.68%, 2002 S2) after its being listed in NHI's drug benefit coverage. Its competitor, the follower COX-2 inhibitor (rofecoxib), although having a disadvantaged diffusion pattern as compared to celecoxib, continuously and steadily increased its market share. Overall, the two new entries took up about 50% (2004 S3) of the market originally held by traditional NSAIDs. This sizable substitution of new drugs for traditional ones shows the potential of a generous health care system in providing better access to new drugs for their beneficiaries by encouraging the adoption and use of expensive medical technology.

The changes of drug expenditure after the market entry of COX-2 inhibitors was very large in Taiwan, the cost of NSAIDs per RA or OA ambulatory visit increased by 40% following the approval of the new drugs. The magnitude of this increase is very high when compared to the overall trend in drug cost increase per ambulatory visit in the NHI system during our study period (drug cost per ambulatory visit NT 220 (yr 2001) and NT 257 (yr 2004), increased by 16.8%) [19]. The Bureau of Taiwan's NHI has tried to manage the potential contribution of new drugs by using reference prices through international comparisons, which require the introductory prices be less than or equal to the median of their list prices in 10 comparator countries (France, Germany, Italy, Sweden, Switzerland, Belgium, Australia, Japan, the United Kingdom, and the United States). However, because of the higher prices of new drugs in the reference countries, which were all developed countries and all had higher per capita gross domestic product (GDP) than that of Taiwan, the reimbursement price of new drugs may be still very high [20]. In our case, two COX-2 inhibitors, celecoxib 100 mg and rofecoxib 25 mg, were both categorized as breakthrough new drugs and reimbursed at 5 to 10 times the price of non-selective NSAIDs per tablet in Taiwan's NHI system. In addition, when prices are set by a regulating agency, the price competition between patented drugs apparently disappears due to the imbalanced market mechanism [21-23]. Although rofecoxib should be considered as a follower in the COX-2 inhibitor category after the introduction of celecoxib in Taiwan's NHI system, its introductory price was twice the introductory price of celecoxib. However, our study found that the high price of rofecoxib did not guarantee a good market share as compared with celecoxib. Besides, the market may become price-insensitive when third parties such as insurance system greatly affect choices on behalf of an individual [3,21-23]. The substitution of nonselective NSAID with higher priced COX-2 inhibitors had thus caused a significant economic impact on the cost of treating chronic pain in patients with RA and OA in whom long-term or life-long consumption of NSAIDs is required. In this situation, the question must be asked whether the increased cost of a new product paralleled its improvement in clinical effectiveness.

The safety of new drugs is another on-going health policy concern, especially when they start being rapidly adopted once they are covered by a national insurance program. There have been several new drugs withdrawn from the market due to severe adverse drug reactions not noticed in the pre-marketing stage. Our study drug, rofecoxib, is one of the most significant examples. The information shock due to rofecoxib's withdrawal was followed by a 6% decline in the cost for RA/OA treatments (NT 304.06 (2004 S3) vs. NT 285.24 (2004 S4)). In the short run, there was only a slight change in drug cost, however. This intervention is more clinically relevant than other ones since the patients' and physicians' perception of the COX-2 inhibitors for RA/OA treatments is expected to be altered. We believed further studies on this issue would be of great benefit to policy makers in managing drug safety signals.

Data and design limitations may affect the extent to which the results of this study can be generalized. Although we have specified three important policy interventions and applied segmented regression analysis for interrupted time series to examine changes of drug expenditure and volume after the implementation of interventions, we could not control unobserved exogenous factors. Therefore, our findings could only present an association rather than a causality effect between implementation of interventions and changes of drug expenditure or utilization. Due to data limitation, we could only evaluate the short-term change after the release of information of drug safety (three months after the withdrawal of rofecoxib). Thus, our aggregated estimate may reveal the change due to the withdrawal event rather than the information on pharmaceutical market. Data were lacking on which to base the differences between reimbursement price and real market price of NSAIDs drugs. As a result, our estimate may not reflect the actual cost and dynamics of the pharmaceutical market. Another limitation is that we assumed that the traditional NSAID and COX-2 inhibitors (new generation of NSAIDs) had identical therapeutic markets, while COX-2 inhibitors may not just be used to replace traditional NSAIDs but they may also be used to increase demand.

## Conclusions

To our knowledge, our study may be the first study to provide a detailed empirical picture of how policy interventions change the drug market. We have found that any correlation between price regulation and a decrease in drug expenditure appeared to be short-lived, especially under the influence of new entry. This study elucidates how new drugs diffuse into the medical care system, how they begin to substitute for existing products, and how they affect the cost of treatment. For policy makers of health insurance program, a scheduled surveillance for each new entry is therefore suggested to provide a cost-effective treatment to their beneficiaries and constrain escalating expenditure.

## Competing interests

The authors declare that they have no competing interests.

## Authors' contributions

WFH, FY H, and YWT were responsible for development of the study concept and design and the preparation of the manuscript. FYH contributed to data acquisition and statistic analysis. All authors participated in the analysis and interpretation of the data of the manuscript. This manuscript has been read and approved by all authors.

## Pre-publication history

The pre-publication history for this paper can be accessed here:

http://www.biomedcentral.com/1472-6963/10/218/prepub
